# High Oxygen Consumption in SARS-COV2: Could the Development of Low-Cost Oxygen Rebreather Be Considered?

**DOI:** 10.3389/fphys.2020.607913

**Published:** 2021-01-21

**Authors:** Nicolas Vallée, Sarah Rives, Anne-Virginie Desruelle, Sebastian Marzetti, Valentin Barchasz, Jean-Jacques Risso, Valentin Gies

**Affiliations:** ^1^Institut de Recherche Biomédicale des Armées_Equipe de Recherche Subaquatique Opérationnelle, Toulon, France; ^2^Université de Toulon, IM2NP, La Garde, France

**Keywords:** rebreather, ventilation, crisis, dioxygen, ICU

Oxygen is a first-line drug in the therapeutic management of patients with SARS-Cov2 (Tobin, 2020). Being limited, oxygen is a resource to save (Maves et al., 2020). In the same time, it should be remembered that medical care can become financially toxic (See and Slonim, 2020) because the cost of healthcare steadily rises (Mycyk, 2020). Is ventilators by lottery (Silva, 2020) the solution? In this way, requiring a low-cost respirator <200€ is attractive (Gies et al., 2020), but its value may be limited in view of the consumption, supply and cost of oxygen. The Swiss newspaper Le Temps reported in its article of July 15, 2020, whose title could be translated as “With Covid-19, the world faces an oxygen shortage,” that the oxygen demand in Italy has increased 10-fold or 14-fold and the costs are likely to soar (Logean, 2020).

We wonder viewing the **High oxygen consumption in SARS-COV2 if the development of low-cost oxygen rebreather could be considered?** The oxygen rebreather, or closed-circuit rebreather, is a simple apparatus well-known to the world of scuba diving and therefore the main goal is to be able to evolve a long time underwater with a small carry of oxygen (Wingelaar et al., 2017). The device is designed so that the majority of the oxygen delivered to the diver is consumed by the organism: the exhaled oxygen must be able to be re-inhaled through a counterlung, while the exhaled carbon dioxide must be able to be extracted using soda lime canister. When the counterlung is empty, the low pressure triggered by the diver's inspiration is enough to activate a regulator connected to a pressurized oxygen cylinder, which replenishes the bladder instantly. Like respirators, one can imagine that a similar device could be added to it in order to limit the loss of oxygen (Figure 1). Since no gas exhaled by the patient is released into the atmosphere, this type of device can help improve the safety of healthcare workers. The development of such a device is simple but it must obviously consider the health safety rules with the use of filters against the virus, but also the rules in force in the field of diving. For example, it is important to properly calibrate the regulator in order to avoid any additional ventilatory effort, which could lead to alveolar damage (Wilmshurst, 2019): electronic systems can easily overcome the defects of the mechanical systems. It is also crucial to have the ability to change soda lime before it is saturated (Arieli, 2008): lime with colored indicators exists. The reaction of soda lime with CO_2_ is exothermic (Shaw and Scott, 1998; Silvanius et al., 2019), but the length of the breathing circuits must be sufficient to dissipate the heat. An ice bath around the soda lime canister could also be suitable. It must be taken into account that the exhaled gases are saturated with water, and that water traps for condensation may be necessary. Additionally, if we are looking to obtain a very high FiO_2_, it is also important to take into account the denitrogenation of the organism (Katz et al., 2017): the nitrogen regularly exhaled can be evacuated by means of regular flushing of the counterlung in the 1st h of treatment. Finally, most of intubated and ventilated COVID patients need levels of positive end-expiratory pressure (PEEP) around 10–15 cm H_2_0 because of their acute respiratory syndrome (Navas-Blanco and Dudaryk, 2020), proportional forces can be applied on the counter lung and the membrane regulator in order to achieve light pressures inside the circuit. These forces can take the appearance of weights, conical springs, or a more complex system synchronized with the respirator. This list is not exhaustive but it retraces the main points that deserve to be raised. This is not insurmountable as divers have been using similar systems for decades.

**Figure 1:**
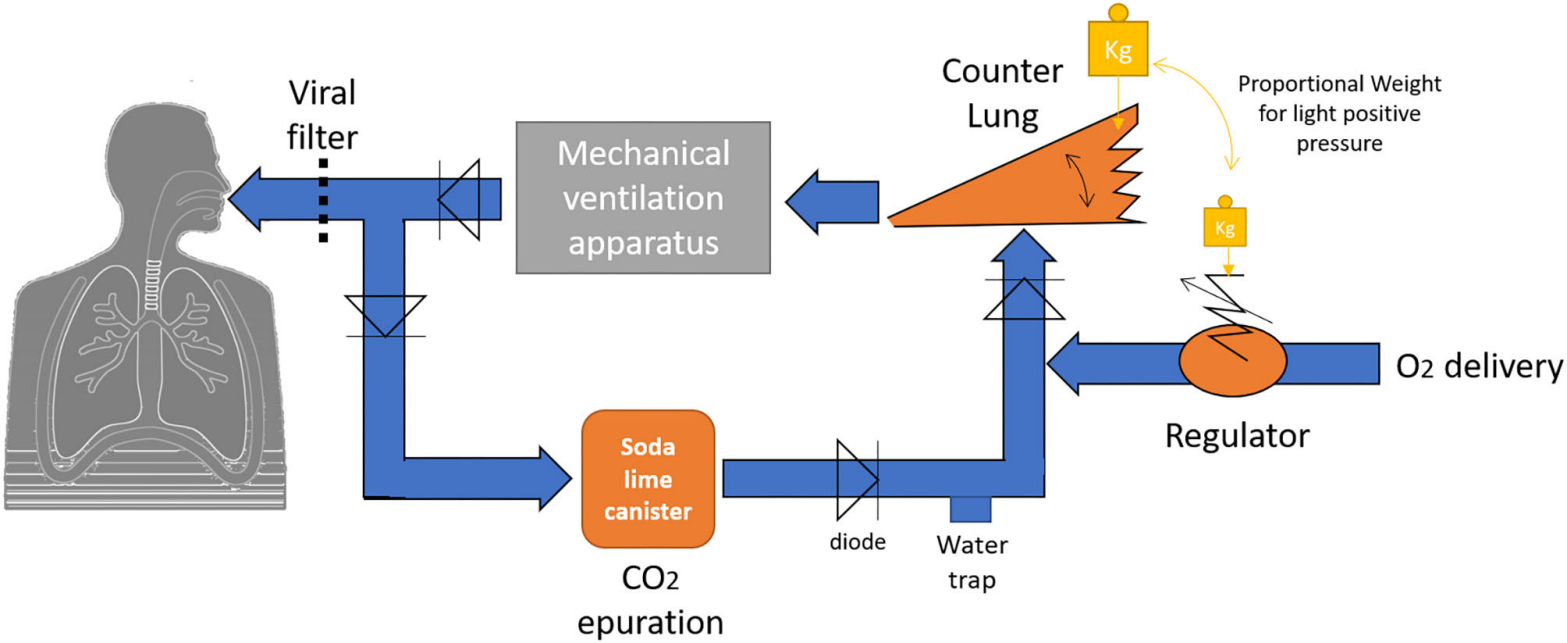
Schematic diagram of a low-cost oxygen rebreather.

## Author Contributions

NV wrote and the other authors proofread and validated the concepts and the manuscript. All authors contributed to the article and approved the submitted version.

## Conflict of Interest

The authors declare that the research was conducted in the absence of any commercial or financial relationships that could be construed as a potential conflict of interest.
